# Establishment of regeneration system of *Peucedanum praeruptorum* and molecular association analysis of coumarin secondary metabolism

**DOI:** 10.3389/fpls.2025.1507930

**Published:** 2025-05-08

**Authors:** Liu Tianliang, Wang Liang, Zhang Yudan, Zheng Yujiao, Zhou Tao, Huang Luqi

**Affiliations:** ^1^ Henan Provincial Ecological Planting Engineering Technology Research Center of Daodi Herbs, School of Pharmacy, Henan University of Chinese Medicine, Zhengzhou, China; ^2^ Research Room of Traditional Chinese Medicine Ethnic Medicinal Materials , Resource Institute for Chinese & Ethnic Materia Medica, Guizhou University of Traditional Chinese Medicine, Guiyang, China; ^3^ State Key Laboratory of Genuine Medicinal Materials, National Resource Center for Chinese Materia Medica, China Academy of Chinese Medical Sciences, Beijing, China

**Keywords:** *Peucedanum praeruptorum* Dunn, coumarin biosynthesis, tissue culture regeneration system, secondary metabolites, phenylpropanoid pathway, gene expression regulation

## Abstract

**Introduction:**

*Peucedanum praeruptorum* is a medicinally critical species whose sustainable utilization is hindered by declining wild populations and insufficient cultivation systems. This study aimed to establish an optimized regeneration system for *P. praeruptorum* and elucidate the molecular mechanisms underlying coumarin biosynthesis, focusing on the roles of key phenylpropanoid pathway genes.

**Methods:**

Leaf, stem, and root explants were cultured on MS media supplemented with varying concentrations of 2,4-D, 6-BA, and IBA to induce callus, proliferation, differentiation, and rooting. Coumarin content (praeruptorins A, B, E) was quantified via HPLC, while RT-qPCR analyzed expression levels of *PpPAL, PpC4H*, and *PpC2'H* genes across tissues.

**Results:**

Optimal media were identified: MS + 0.5 mg/L 2,4-D + 0.5 mg/L 6-BA (callus induction, 85.71% efficiency), MS + 1.0 mg/L 6-BA + 0.5 mg/L IBA (proliferation), and MS + 0.5 mg/L IBA (rooting, 69.44% success). Adventitious buds exhibited the highest total coumarin content (3.67× callus), with roots of seedlings accumulating 1.67× more coumarin than leaves. Expression of PpC2'H dominated across materials (*PpC2'H > PpC4H > PpPAL*), correlating strongly with coumarin levels.

**Discussion:**

The study demonstrates that hormonal crosstalk (auxin-cytokinin balance) critically regulates morphogenesis, while *PpC2'H* acts as a bottleneck gene for coumarin synthesis. These findings enable targeted metabolic engineering (e.g., *PpC2'H* overexpression) to enhance yields, offering a sustainable alternative to wild harvesting.

## 1 Introduction

*Peucedanum praeruptorum* is a member of the Umbelliferae plant family and has been used as a root medicine for centuries. The annual demand for *P. praeruptorum* is more than 3,000 tons. There are hundreds of traditional Chinese medicine prescriptions and Chinese patented medicines that utilize *P. praeruptorum* as a raw medicinal material and represents the initial product used by numerous pharmaceutical companies (Liu et al., 2022). *P. praeruptorum* is widely distributed in the central, southern, and eastern regions of China, including Zhejiang (Chunqianhu), Anhui (Ningqianhu) and Jiangxi (Xinqianhu). Coumarins are recognized as the main characteristic active ingredients of *P. praeruptorum.* Among them, pyranocoumarin, as the representative, not only has a long history of application in traditional medicine, but also shows great potential in modern drug research and development, covering cardiovascular, respiratory, anti-tumor, anti-inflammatory and other fields. From 2019 to 2021, the quality of *P. praeruptorum* was deteriorating. The results of national drug sampling inspection demonstrated that Qianhu was severely substandard (Liu et al., 2021; Guo et al., 2021; Jing et al., 2023). Furthermore, there is currently no industrially cultivated variety of *P. praeruptorum* and imitation wild cultivation technology has several problems, including those related to heavy metals, pests and diseases (Xiang, 2006; Li and Zhang, 2014; Du et al., 2021).

The utilization of plant tissue culture technology to produce and prepare secondary metabolites of medicinal plants is a key strategy for the sustainable development and utilization of Chinese herbal medicine resources. Primarily, the construction of a genetic transformation system using this technology is a basis for genetic transformation of medicinal plants. Commonly used receptors include embryogenic callus, suspension cells, and protoplasts. Among these, callus has a high transformation efficiency and a fast growth rate, which makes it an excellent choice for rapid verification of gene function (Zhang et al., 2023). Additionally, this technology is not limited by time and space, since a large number of seedlings can be produced in limited space. This approach protects plants from plant viruses and maintains their desired traits. Moreover, the rapid propagation of plants using this technology allows for the extraction of more secondary metabolites for scientific research or clinical purposes (Guo et al., 2016). Therefore, it is of great importance to establish a stable and efficient callus regeneration system for the study of genetic transformation and the sustainable utilization of resources.

Research on the tissue culture regeneration system of *P. praeruptorum* is still in its infancy (Wu, 2017; Li, 2022). The present study sets out to address two fundamental questions. Firstly, it seeks to ascertain whether coumarin production is accompanied by root or leaf differentiation. Secondly, it aims to identify the genes in the phenylpropane synthesis pathway that are most critical for coumarin synthesis. The prevailing view is that coumarin is distributed through the roots and transported upwards in the prehensile. However, the hypothesis put forward in this study is that coumarin is synthesized *in situ* in a variety of tissues. Furthermore, we hypothesize that the key determinants of coumarin content are downstream genes such as *C2’H*, rather than upstream genes such as *PAL*. The establishment of a successful regeneration system will facilitate a deeper understanding of these issues, which will in turn contribute to the controlled production of coumarin in an artificial environment.

## 2 Materials and methods

### 2.1 Induction of callus

We cut 1 cm long segments of leaves, stems, and roots of *P. praeruptorum* and treated them with a 75% ethanol solution for 30 s followed by a 0.1% HgCl_2_ disinfection solution for 10 min. Plant material was then inoculated within Murashige and Skoog (MS) medium supplemented at 5 different concentrations of 2,4-D (0, 0.5, 1.0 mg/L) and 6-BA (0, 0.5, 1.0 mg/L). Each dish was inoculated with 7 explants, including 5 dishes in each group. After 14 d, the callus induction was observed and the callus induction rate for each group was calculated.

### 2.2 Proliferation and differentiation of callus

The callus of *P. praeruptorum* was cut into blocks of approximately 0.3 cubic cm and inoculated on an MS medium supplemented with 5 different concentrations of IBA (0, 0.5, 1.0 mg/L) and 6-BA (0, 0.5, 1.0 mg/L). Each bottle was inoculated with 6 callus clumps, 8 bottles in each group. After 28 d, the growth status of callus, the color and texture of callus, the number and morphology of adventitious buds, and the proliferation rate were observed and recorded. The differentiation rate and browning rate of each bottle were also calculated.

### 2.3 Rooting of adventitious buds

Adventitious buds of *P. praeruptorum* approximately 3 cm in length and 2–3 leaves were inoculated on MS medium containing 5 different concentrations of IBA (0.2, 0.4, 0.6, 0.8, 1.0 mg/L). Each bottle was inoculated with 6 adventitious buds, 8 bottles in each group. After 14 d, the growth status of adventitious buds, the number and morphology of rooting of multiple buds, and the browning rate were observed and recorded. The rooting rate and average rooting number of each group were also calculated.

### 2.4 Transplanting of clustered seedlings

The cluster seedlings of *P. praeruptorum* with root length of more than 3 cm were selected and acclimated for 14 d on the filter paper bridge of MS liquid medium and the mixed nursery soil of humus soil: vermiculite: perlite = 2: 1: 1. 3 cluster seedlings were inoculated in each bottle/pot, with a total of 16 bottles/pot in each group. After 14 d, the growth status of tissue culture seedlings, the morphology, and the color of leaves and roots were observed and recorded. The survival rate of each group was calculated.

### 2.5 Determination of coumarin content

The sample solution was created by using 0.1 g of sample powder mixed with 5 ml of chloroform. The solution was exposed to ultrasonic treatment for 30 min and followed by extraction of 4 mL of continuous filtrate. The resulting material was dried using a nitrogen blowing instrument, subsequently dissolved using 1 mL of methanol, and then filtered (0.22 μm) before analysis. The chromatographic column used was a Symmetry C18 (4.6 * 250 mm, 5 μm). The mobile phase was methanol-water (80∶ 20). The injection volume was 10 μL, the detection wavelength was set at 321 nm, the detection time was set at 15 min, the flow rate was set at 1.0 mL/min, and the column temperature was set at 30°C. Praeruptorin A was quantified at 60, 80, 100, 200, 400 μg/mL, Praeruptorin B and Praeruptorin E were quantified at 10, 20, 40, 60, 80 μg/mL. The standard curves were *Y* = 23.805*X*-451.350 (*R^2^* = 0.9994), *Y* = 21.244*X*-43.189 (*R^2^* = 0.9995), *Y* = 19.886*X*-36.769 (*R^2^* = 0.9994).

### 2.6 Real-time quantitative PCR

The universal premix of SYBR Green I chimeric fluorescence method (Vazyme, Nanjing, China) was used. The final concentration of each primer was 0.2 μM (**Supplementary Table S1**). The cDNA of 10 ng total RNA was used as the template, and the total system was 20 uL. Real Time PCR reaction was performed on the ABI 7500 machine with a two-step method. The reaction procedure was 95°C/10 s, and 40 cycles of 95°C/10 s and 60°C/30 s. The data were exported, and the 2^^-(ΔCt)^ value was calculated.

## 3 Results

### 3.1 Effects of different hormone combinations on callus induction

**Figure 1:**
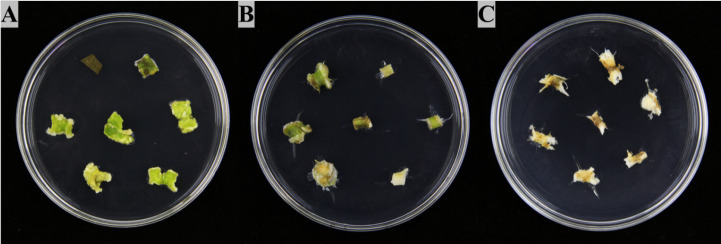
Induction of different explants of *P. praeruptorum*. **(A)** Group 3 Leaf (**Supplementary Table S2**), **(B)** Group 3 Stem (**Supplementary Table S2**), **(C)** Group 3 Root (**Supplementary Table S2**).

Explants of *P. praeruptorum* could successfully induce callus in the medium with different ratios of 2,4-D to 6-BA, however, the ability of callus induction in leaves, stems, and roots were significantly different (**Supplementary Table S2**). In addition, the morphological characteristics of callus induced by different explants of *P. praeruptorum* also varied. Among them, leaf calluses were mostly light white or yellow-green and comprised of loose and fragile callus blocks gathered in granular shape. Callus derived from stem segments was mostly yellow-green and composed of dumbbell-shaped, compact structures. The root callus was light yellow or brown forming a loose and tight callus block. At a 2,4-D/6-BA concentration ratio of 1 (**Figure 1**), the leaves, stems and roots of different explants of *P. praeruptorum* were inoculated on the medium. After two weeks of dark culture, callus began to form around the wound. The induction rate was the highest compared with other groups, which were 85.71%, 51.43% and 20.00%, respectively. Overall, leaves were determined to be the best explant selection for *P. praeruptorum* and MS + 2,4-D 0.5 mg/L + 6-BA 0.5 mg/L was the optimal induction medium for callus of *P. praeruptorum*.

### 3.2 Effects of different hormone ratios on callus proliferation and differentiation

**Figure 2:**
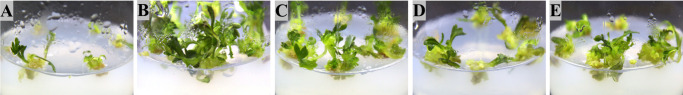
Growth status of callus of *P. praeruptorum*. **(A)** Group 1 (**Supplementary Table S3**), **(B)** Group 2 (**Supplementary Table S3**), **(C)** Group 3 (**Supplementary Table S3**), **(D)** Group 4 (**Supplementary Table S3**), **(E)** Group 5 (**Supplementary Table S3**).

A reduction in the concentration ratio of IBA to 6-BA in the medium resulted in a decrease in the proliferation rate of callus, which subsequently increased. In contrast, the differentiation rate of callus exhibited an inverse relationship (**Supplementary Table S3**). At a IBA/6-BA concentration ratio of 2 (**Figure 2A**), the callus tended to proliferate, exhibiting the highest growth state and the most advanced browning situation among all groups. When the IBA/6-BA concentration ratio was 1 (**Figure 2C**), the callus tended to differentiate, the number of cluster buds were relatively large, and the size was suitable and not intertwined. Furthermore, the color of the leaves was dark green, the growth was consistent, and the differentiation rate was 68.75%. At a IBA/6-BA concentration ratio of 0.5 (**Figure 2E**), the callus exhibited a bias towards proliferation, with an overall green color, a relatively compact structure, and the production of embryonic cells, and the proliferation rate was 66.67%. Furthermore, when the MS medium contained solely IBA (**Figure 2B**), the bud points exhibited differentiation, browning, and a generalized growth state. In contrast, when the MS medium contained solely 6-BA (**Figure 2D**), the overall growth state of the callus was poor, and browning was pronounced. Overall, MS + 6-BA 1.0 mg/L + IBA 0.5 mg/L was determined as the optimal proliferation medium for callus and MS + 6-BA 0.5 mg/L + IBA 0.5 mg/L was the most suitable differentiation medium for cluster buds of *P. praeruptorum*.

### 3.3 Effects of different hormone concentrations on rooting of adventitious buds

**Figure 3:**
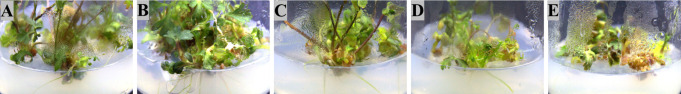
Rooting situation of clustered plantlets of *P. praeruptorum*. **(A)** Group 1 (**Supplementary Table S4**), **(B)** Group 2 (**Supplementary Table S4**), **(C)** Group 3 (**Supplementary Table S4**), **(D)** Group 4 (**Supplementary Table S4**), **(E)** Group 5 (**Supplementary Table S4**).

As the concentration of IBA in the medium increased gradually, the rooting rate of adventitious buds initially increased and then subsequently decreased (**Supplementary Table S4**). At a concentration of 0.2 mg/L (**Figure 3A**), the number of rooting of cluster buds was found to be the lowest among all groups, with the majority exhibiting fibrous roots, which were fine and short in shape. At a concentration of 0.8 mg/L (**Figure 3D**), the growth state was relatively poor and exhibited a limited number of rooting buds. However, the main root was larger than the fibrous root. Upon reaching a concentration of 1.0 mg/L (**Figure 3E**), the growth state was found to be the most adverse among all groups. This was accompanied by a notable inhibition of rooting of cluster buds and a significant browning phenomenon. Furthermore, when the IBA concentration was between 0.4 and 0.6 mg/L (**Figures 3B, C**), the growth state of cluster buds was the most optimal among all groups. The rooting rate was also the highest among groups, reaching 69.44% and 66.67%, respectively. The rooting rate, average rooting number, and growth state of multiple shoots were analyzed to determine the optimal rooting medium for the tissue culture seedlings. The results indicated that the IBA concentration of 0.5 mg/L, equivalent to MS + IBA 0.5 mg/L, was the most effective for rooting the seedlings.

### 3.4 Effects of different culture substrates on cluster seedlings hardening

**Figure 4:**
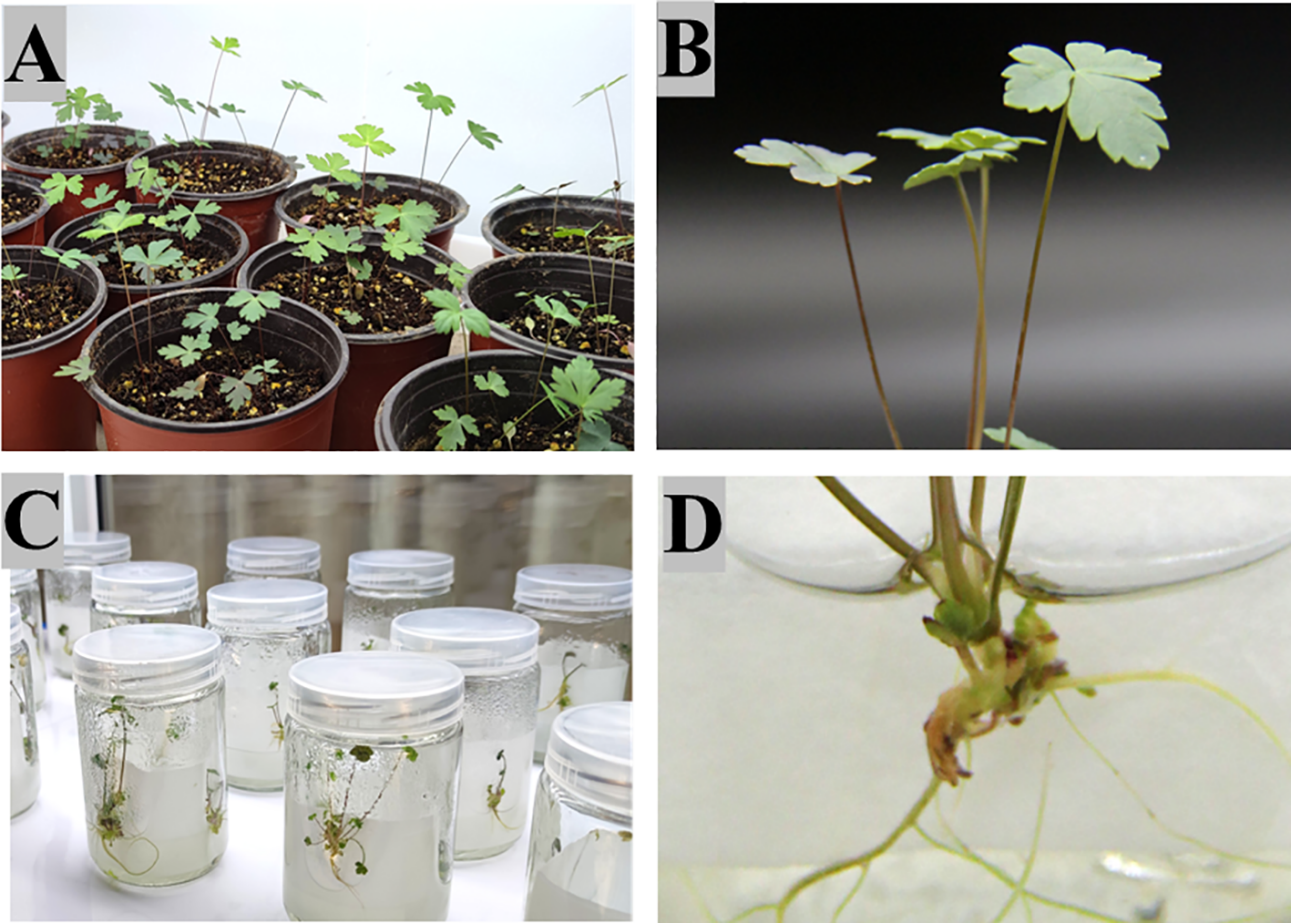
Refining of aseptic seedlings of *P. praeruptorum*. **(A, B)** Group 1 (**Supplementary Table S5**), **(C, D)** Group 2 (**Supplementary Table S5**).

The results demonstrated that the survival rate of seedlings grown in humus soil, vermiculite, and perlite in a 2: 1: 1 ratio was 79.16%. The survival rate of seedlings grown in MS liquid medium using the filter paper bridge method was higher, reaching 91.67% (**Supplementary Table S5**). However, when considering the growth status of the tissue culture seedlings, it can be observed that those grown in the nursery soil (**Figures 4A, B**) exhibited relatively good growth, with the leaves stretched and a dark green color. In contrast, those grown on the filter paper bridge (**Figures 4C, D**) exhibited relatively poor growth, with the leaf shape being shrunken and the color being light green. However, subsequent observation of root growth revealed that the root expansion and growth rate of tissue culture seedlings on the filter paper bridge were significantly greater than those observed in the seedling soil.

### 3.5 Differences in secondary metabolites of coumarin in different material

**Figure 5:**
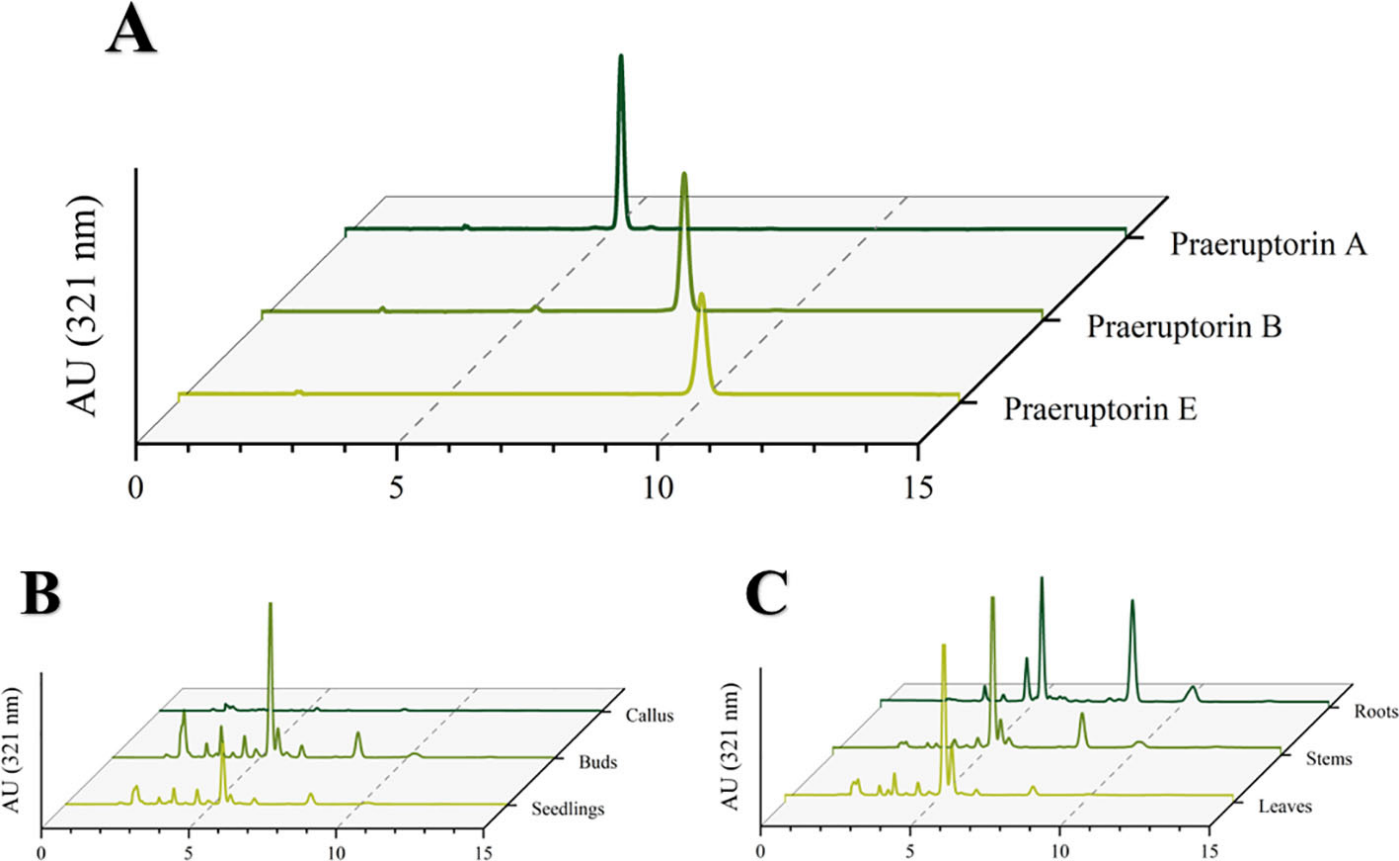
**(A)** HPLC chromatogram of coumarin standard of *P. praeruptorum;* **(B)** HPLC chromatogram of different tissue culture materials of *P. praeruptorum*; **(C)** HPLC chromatogram of different parts of tissue culture seedlings of *P. praeruptorum*.

**Figure 6:**
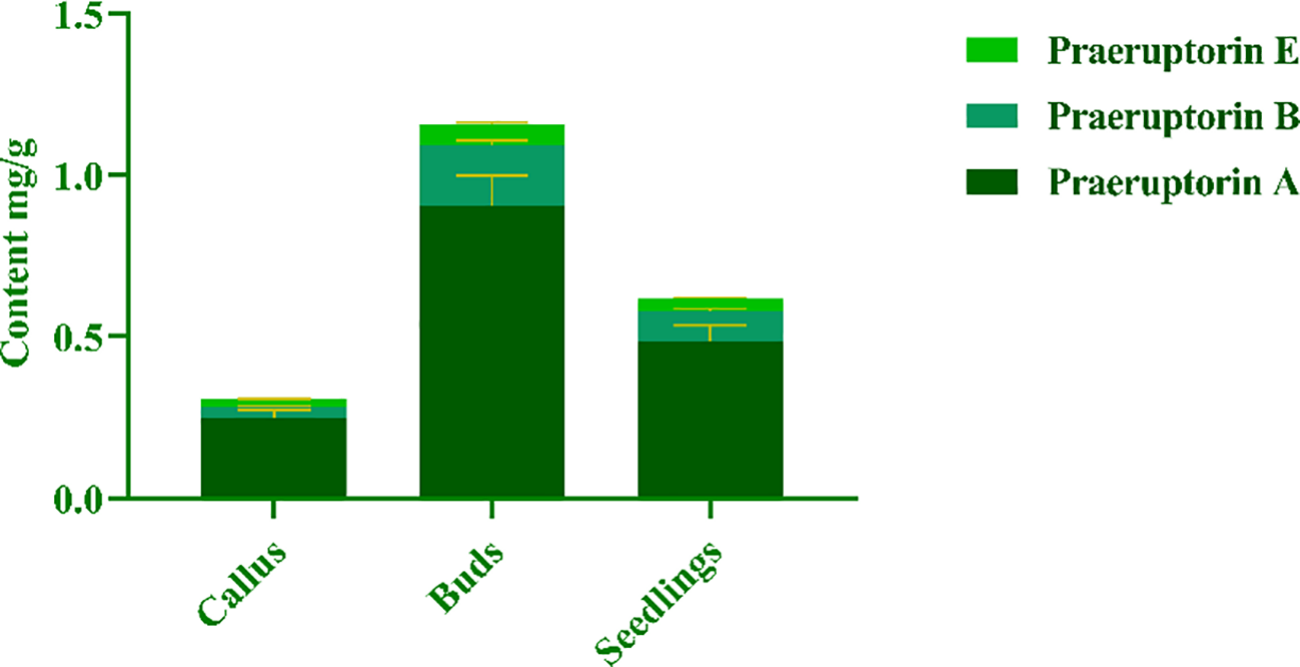
Total coumarin content of different tissue culture materials of *P. praeruptorum*.

**Figure 7:**
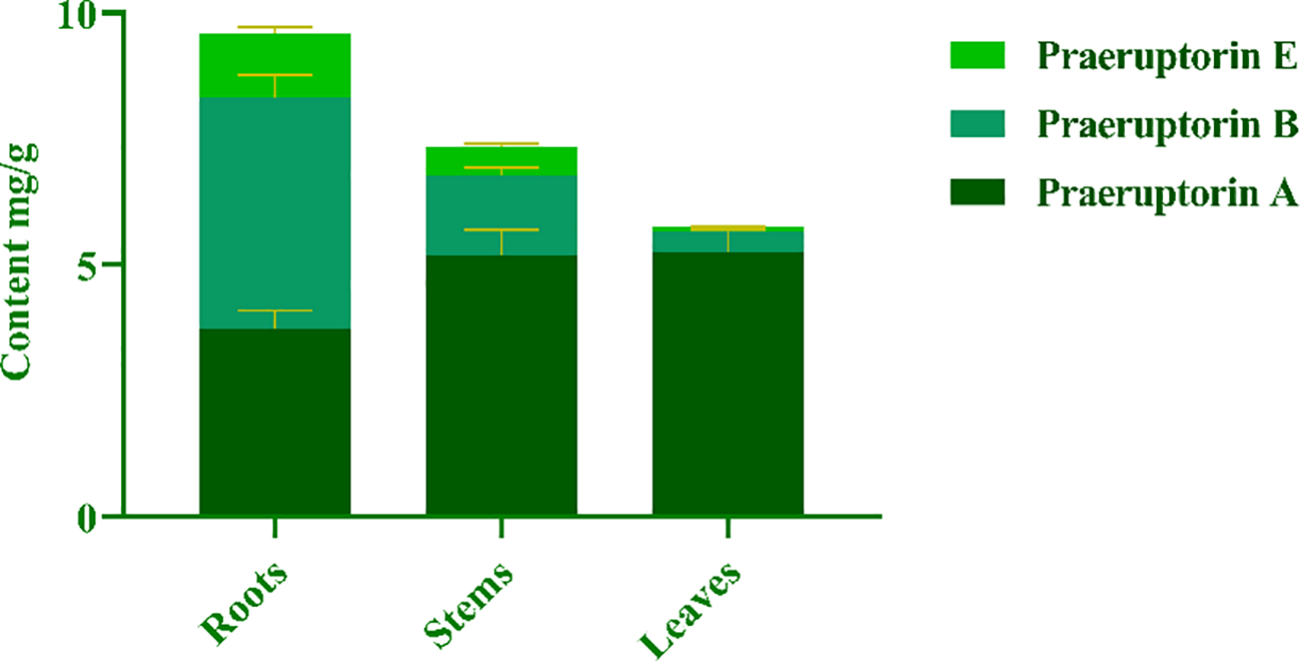
Total coumarin content in different parts of *P. praeruptorum* tissue culture seedling.

The purpose of this experiment was to determine the contents of three representative coumarins (Praeruptorin A, Praeruptorin B and Praeruptorin E) in different tissue culture materials (callus, adventitious buds and cluster seedlings). Additionally, the contents of these three coumarins in different parts (roots, stems and leaves) of tissue culture seedlings were also determined (**Figure 5**). The total content of coumarin in different materials was analyzed. We found that the total coumarin content, from highest to lowest, was as follows: adventitious buds > cluster seedlings > callus (**Figure 6**). Furthermore, the total coumarin content in adventitious buds was 3.67 times that of callus. The total content of coumarin was highest in the roots of the tissue culture seedlings and 1.67 times more than in the leaves (**Figure 7**). Moreover, the content of coumarins in different tissue culture materials was as follows: Praeruptorin A > B > E. Furthermore, the content of Praeruptorin A was found to be an important factor in determining the total content of coumarins. In different parts of tissue culture seedlings, the content of Praeruptorin A was found to be higher in stems and leaves, whereas the contents of Praeruptorin B and Praeruptorin E were found to be higher in roots than in stems and leaves.

### 3.6 Differences in key genes for coumarin synthesis in different materials

**Figure 8:**
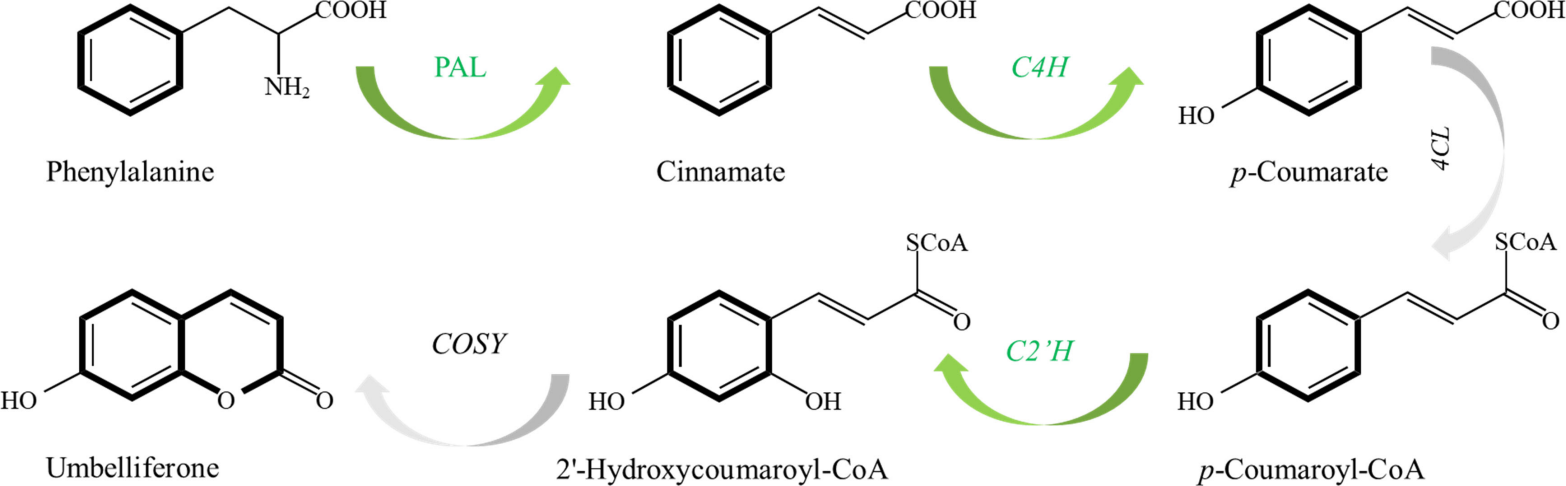
The proposed biosynthetic pathways of coumarins in *P. praeruptorum*.

**Figure 9:**
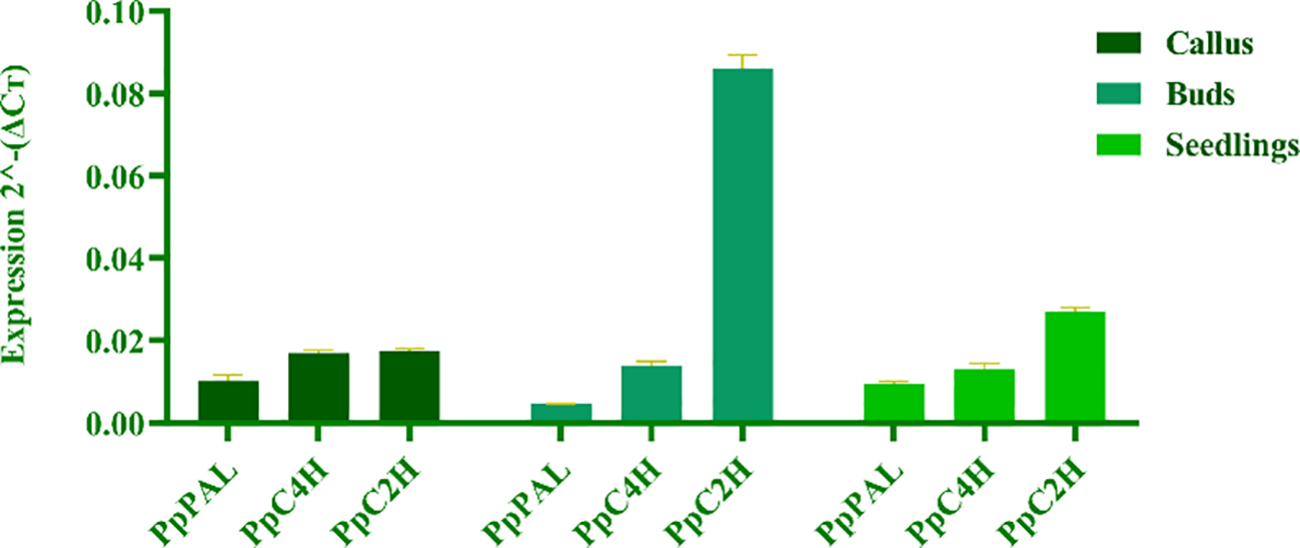
Coumarin synthesis key enzyme gene expression quantity of different tissue culture materials of *P. praeruptorum*.

**Figure 10:**
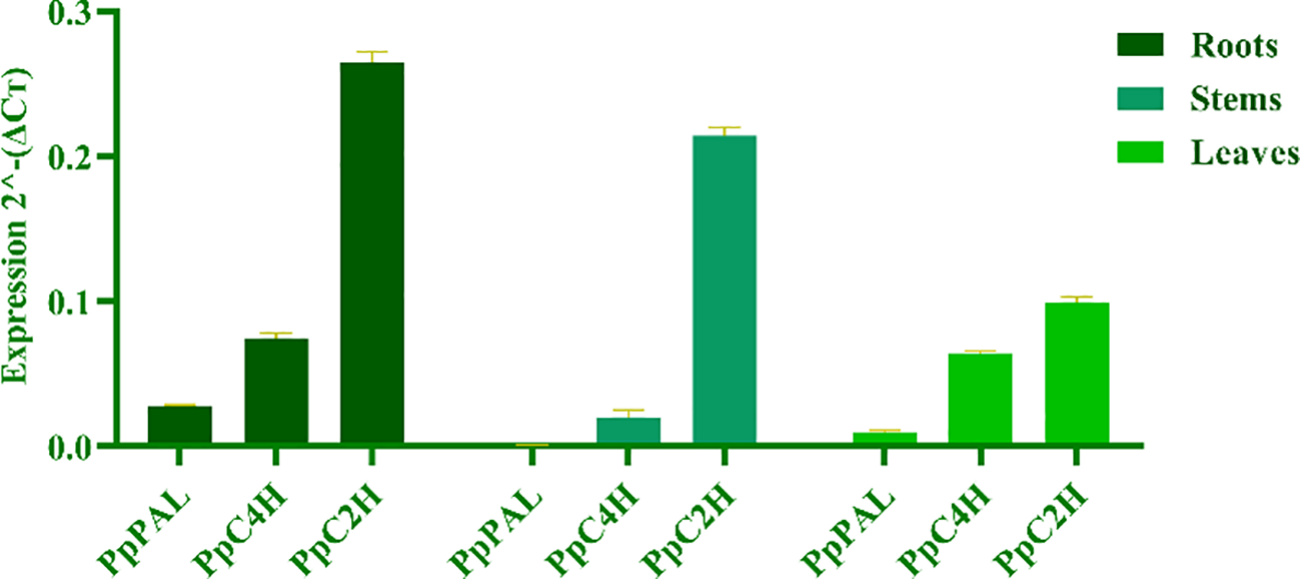
Coumarin synthesis key enzyme gene expression quantity in different parts of *P. praeruptorum* tissue culture seedlings.

To reveal the differences of coumarin secondary metabolites in different materials of *P. praeruptorum* at the molecular level, we determined the expression levels of key enzyme genes *PpPAL*, *PpC4H*, *PpC2’H* in different tissue culture materials (i.e., embryonic callus, adventitious buds, cluster seedlings) and different parts (i.e., roots, stems, leaves) of tissue culture seedlings (**Figure 8**). The results showed that the expression level of *PpC2’H* was higher than that of *PpC4H* and *PpPAL* in different experimental materials or different tissues (**Figure 9**). The expression level of *PpC2’H* in adventitious buds was much higher than that in cluster seedlings and callus. This observation was posited as one of the reasons why the total coumarin content was higher than the other two groups. In addition, the expression trend of the three key enzyme genes in different parts of the tissue culture seedlings was also *PpC2’H* > *PpC4H* > *PpPAL* (**Figure 10**). This was consistent with the result that the total content of coumarin in roots was higher, indicating that the expression of *PpC2’H* was positively correlated with the total content of coumarin in *P. praeruptorum*.

## 4 Discussion

The efficacy of medicinal plant tissue culture systems hinges on the synergistic interplay of explant selection, culture conditions, and hormonal regulation. Our findings underscore the critical role of auxin-cytokinin crosstalk in steering the developmental fate of *P. praeruptorum* callus (Guo et al., 2016). Specifically, 2,4-D emerged as indispensable for callus induction, likely due to its strong auxin-like activity in triggering dedifferentiation and cell proliferation, as observed in other Apiaceae species (Zhang et al., 2023). The antagonistic relationship between 6-BA and IBA/NAA reflects a conserved hormonal mechanism governing plant morphogenesis. This duality also highlights the need for stage-specific hormonal optimization to maximize biomass yield and secondary metabolite production. Previous studies have employed the combination of NAA and 6-BA for experimentation (Wu, 2017; Li, 2022). However, preliminary experiments conducted on this basis revealed that the cluster buds of *P. praeruptorum* were unable to take root on MS medium with different ratios of NAA to 6-BA. Interestingly, the superior rooting efficiency of IBA over NAA aligns with its stability and reduced cytotoxicity in promoting adventitious root formation, a phenomenon corroborated in Bupleuri Radix (Bonnie et al., 2001; Dong et al., 2024; Li, 2020).

The transfer of tissue culture seedlings from heterotrophic to autotrophic life is a crucial step in the process of acclimation. Seedlings must undergo a conditioning process that gradually adapts them to their new environment before their plant organs fail to perform their functions. This process is essential for the survival and growth of tissue culture seedlings. The filter paper bridge method significantly enhanced root vigor and transplant survival rates, outperforming traditional soil substrates. It can be hypothesized that this system will facilitate continuous nutrient uptake while minimizing mechanical stress on delicate roots. Previous studies have demonstrated the efficacy of this method in promoting the growth of roots, stems, leaves, organ fresh weight, and dry weight (Felix et al., 2016; Blidar et al., 2012; Sun et al., 2002). The present study demonstrates that the root system of tissue culture seedlings using the filter paper bridge method exhibits rapid expansion and growth, rendering them more suitable for the establishment of hairy root genetic systems, structural gene function verification, transcriptional regulation hormone treatment, and other related molecular pharmacognosy experiments.

As coumarin components mainly accumulate in roots, the prevailing view is that there is a mechanism of root-to-leaf transport and regulation of coumarin in *P. praeruptorum* (Li X. L. et al., 2022; Yu et al., 2013). Coumarin dynamics across tissues challenge the conventional “root-centric” synthesis paradigm. While roots exhibited the highest post-transplant coumarin content, adventitious buds—devoid of roots—accumulated 3.67-fold more coumarin than callus, implying *de novo* synthesis in aerial tissues. This spatial-temporal pattern aligns with the phased expression of biosynthetic genes: *PpC2’H* dominated in undifferentiated callus and adventitious buds, whereas its expression declined in rooted seedlings as metabolic priorities shifted to vegetative growth. The strong correlation between *PpC2’H* expression and total coumarin content positions this gene as a bottleneck in the phenylpropanoid pathway. Unlike upstream genes (*PpPAL, PpC4H*) that channel precursors into competing branches, *PpC2’H* directly catalyzes umbelliferone formation—the keystone intermediate for pyranocoumarins like praeruptorins. This substrate specificity explains its outsized influence on coumarin yield.

To harness these insights, we propose a dual biotechnological strategy: (1) CRISPR/Cas9-mediated knockout of PpCHS (chalcone synthase) to divert flux from flavonoids toward coumarins, and (2) Agrobacterium-mediated overexpression of *PpC2’H* under a dexamethasone-inducible promoter for temporally controlled synthesis. Concurrently, elicitation with methyl jasmonate—a proven inducer of phenylpropanoid pathways—could further amplify yields in bioreactor-cultured callus. Such approaches would address the current reliance on wild-harvested *P. praeruptorum*, which faces sustainability crises due to habitat loss and overexploitation. Limitations of this study include the absence of transcriptomic validation and single-timepoint metabolite sampling. Longitudinal tracking of gene expression alongside coumarin accumulation could refine phase-specific engineering targets. Additionally, in planta RNAi silencing of competing pathway genes (e.g., PpANS for anthocyanins) may further optimize metabolic flux. These directions, coupled with field trials of tissue-cultured lines, will bridge the gap between laboratory findings and industrial-scale coumarin production.

## Data Availability

The datasets presented in this study can be found in online repositories. The names of the repository/repositories and accession number(s) can be found in the article/**Supplementary Material**.

## Supplementary material

Supplementary Table 1 Real-time fluorescent quantitative PCR primers of *P. praeruptorum.*

Supplementary Table 2 Effects of different hormone ratios on the induction of *P. praeruptorum*.

Supplementary Table 3 Effects of different hormone ratios on growth status of callus of *P. praeruptorum*.

Supplementary Table 4 Effects of different hormone concentrations on rooting rate of cluster buds of *P. praeruptorum*.

Supplementary Table 5 Effects of different culture substrates on hardening of aseptic seedlings of *P. praeruptorum*.
