# Supplementary material for: Establishment of regeneration system of Peucedanum praeruptorum and molecular association analysis of coumarin secondary metabolism
Source: Front Plant Sci. 2025 May 8;16:1507930. doi: 10.3389/fpls.2025.1507930 (PMC12095164; doi:10.3389/fpls.2025.1507930)
Supplement: Supplementary Table 1 — Real-time fluorescent quantitative PCR primers of P. praeruptorum. [file Table1.doc]

## Supplementary Tables

Table 1 Real-time fluorescent quantitative PCR primers of *P. praeruptorum*

| Gene | Forward primer | Reverse primer |
| --- | --- | --- |
| *PpSAND* | ACAGAAGAGCCTCATGAATC | CAAGCAAAGGCGTCATATCA |
| *PpPAL* | CGAGATAGCAGCCACCTGAG | TAATGGTACCACCAACGGGC |
| *PpC4H* | TAGACACTCCAGGAGGAGGC | GGTCAATGCATGGTGGCTTG |
| *PpCHS* | CGATTTGTCACACATGCGCT | TGTGTCGACCAAAGCACGTA |
| *PpC2’H* | TCAGAATGGCGTCCAACTCC | TCAAAGCCCTTATGCAGGGG |

Table 2 Effects of different hormone ratios on the induction of *P. praeruptorum*

| Group | 2,4-D  mg/L | 6-BA  mg/L | Induction rate | | |
| --- | --- | --- | --- | --- | --- |
|  |  |  | Leaf | Stem | Root |
| 1 | 1.0 | 0.5 | 80.00 ± 11.43 | 48.57 ± 19.38 | 20.00 ± 14.57 |
| 2 | 1.0 | 0.0 | 37.14 ± 14.57 | 22.85 ± 7.00 | 8.57 ± 7.00 |
| 3 | 0.5 | 0.5 | 85.71 ± 15.65 | 51.43 ± 14.57 | 20.00 ± 7.00 |
| 4 | 0.0 | 1.0 | 22.86 ± 14.57 | 14.29 ± 9.04 | 5.71 ± 7.00 |
| 5 | 0.5 | 1.0 | 71.43 ± 9.04 | 42.86 ± 9.04 | 17.14 ± 5.71 |

Table 3 Effects of different hormone ratios on growth status of callus of *P. praeruptorum*

| Group | IBA mg/L | 6-BA mg/L | Proliferation rate | Differentiation rate | Growth state |
| --- | --- | --- | --- | --- | --- |
| 1 | 1.0 | 0.5 | 25.00 ± 8.33 | 29.17 ± 7.22 | + |
| 2 | 1.0 | 0.0 | 12.50 ± 7.22 | 72.92 ± 8.07 | +++ |
| 3 | 0.5 | 0.5 | 14.58 ± 5.51 | 68.75 ± 5.51 | +++++ |
| 4 | 0.0 | 1.0 | 29.17 ± 7.22 | 41.67 ± 8.33 | ++ |
| 5 | 0.5 | 1.0 | 66.67 ± 8.33 | 20.83 ± 7.22 | +++++ |

Table 4 Effects of different hormone concentrations on rooting rate of cluster buds of *P. praeruptorum*

| Group | IBA mg/L | Rooting rate | Growth state |
| --- | --- | --- | --- |
| 1 | 0.2 | 39.58 ± 8.07 | ++ |
| 2 | 0.4 | 68.75 ± 5.51 | ++++ |
| 3 | 0.6 | 66.67 ± 8.33 | ++++ |
| 4 | 0.8 | 72.92 ± 8.07 | ++ |
| 5 | 1.0 | 27.08 ± 11.60 | + |

Table 5 Effects of different culture substrates on hardening of aseptic seedlings of *P. praeruptorum*

| Group | Substrate | Survival rate | Growth state |
| --- | --- | --- | --- |
| 1 | Seedling soil | 79.17 ± 16.14 | +++++ |
| 2 | Filter paper bridge | 91.67 ± 14.43 | ++++ |
